# Smoking-attributable mortality in South America: A systematic review

**DOI:** 10.7189/jogh.11.04014

**Published:** 2021-03-27

**Authors:** Alexandra Giraldo-Osorio, Mónica Pérez-Ríos, Julia Rey-Brandariz, Leonor Varela-Lema, Agustín Montes, Adriana Rodríguez-R, Nerea Mourino, Alberto Ruano-Ravina

**Affiliations:** 1 Department of Preventive Medicine and Public Health, University of Santiago de Compostela, Santiago de Compostela, Spain; 2 Department of Public Health, Health Promotion and Disease Prevention Research Group (Grupo de Investigación Promoción de la Salud y Prevención de la Enfermedad – GIPSPE), Universidad de Caldas, Manizales, Colombia; 3 Carolina Foundation, Madrid, Spain; 4 Consortium for Biomedical Research in Epidemiology & Public Health (CIBER en Epidemiología y Salud Pública – CIBERESP), Madrid, Spain

## Abstract

**Background:**

Dating from the 1920s and linked to the increase in mortality among smokers, tobacco has become one of the most studied health risk factors. Tobacco-use series, whether for the general population or for specific groups, are unavailable for most South American countries, something that hinders the characterisation of this risk factor.

**Objectives:**

To identify and analyse studies that estimate smoking-attributable mortality (SAM) in South America and provide an overview of the impact of smoking habit on mortality in the region.

**Methods:**

Systematic review using PubMed, Embase, LILACS, *Biblioteca Virtual en Salud*, Google Scholar and Google, and including all papers published until June 2020 reporting studies in which SAM was estimated.

**Results:**

The search yielded 140 papers, 17 of which fulfilled the inclusion criteria. There were SAM estimates for all South American countries, with Argentina having the most. The first estimate covered 1981 and the latest, 2013. The method most used was prevalence-based. Regardless of the country and point in time covered by the estimate, the highest figures were recorded for men in all cases. The burden of attributable vs observed mortality varied among countries, reaching a figure of 20.3% in Argentina in 1986. The highest SAM burden was registered for the group of cardiovascular diseases.

**Conclusions:**

SAM estimates are available for all South American countries but the respective study periods differ and the frequency of the estimates is unclear. For 4 countries, the only estimates available are drawn from reports, something that does not allow for a detailed assessment of the estimates obtained. To help with decision-making targeted at evaluating and enhancing the impact of smoking control policies, further studies are needed in order to update the impact of smoking on all countries across South America.

Dating from the 1920s and linked to the increase in mortality among smokers, tobacco has become one of the most studied health risk factors [1]. The characterization of the epidemiology of smoking in any given population is based both on the study of the prevalence of tobacco use and related characteristics, and on the estimate of tobacco’s impact on mortality. It is estimated that worldwide some 942 million men and 172 million women over the age of 15 years smoke cigarettes [2]. Population-based studies on the prevalence of and trends in tobacco use are frequent in high-income countries, where, following one of the strategies included in the MPOWER (Monitor, Protect, Offer, Warn, Enforce, Raise) measures, tobacco monitoring or surveillance systems have been implemented [3]. Tobacco-use series, whether for the general population or for specific groups, are unavailable for most South American countries, something that hinders the characterisation of this risk factor [2]. Furthermore, it must be borne in mind that prevalences vary widely across the continent, eg, ranging from an estimated 38.7% in Chile to 7.4% in Ecuador [4].

To form an objective idea of the influence exerted by tobacco use on total mortality, attributable mortality (AM) is estimated. Establishment of the causal relationship between tobacco use and mortality shifts over time, with tobacco currently being causally associated with 22 disease subgroups or individual nosological disease entities [5]. AM is a valuable indicator with which the progress of the smoking epidemic can be analysed from a standpoint parallel to that of prevalence, and the impact of tobacco use on population health can be measured [6]. Rather than being directly based on the use of death certificates, this estimate relies instead on the use of epidemiological methods that are based on the acceptance of different assumptions [7]. The methods most frequently used can be generally classified into prevalence-based and non-prevalence-based. Whereas the application of a prevalence-based method is subject to knowledge of the prevalence of smoking in a given population, application of a non-prevalence-based method is dependent on lung cancer mortality rates [7].

Assessing the availability of smoking-attributable mortality (SAM) estimates for the countries of South America would help form a general overview of this public health problem, an indispensable aspect for the planning of health programmes and policies [7]. Accordingly, the twin aims of this study were: to identify and analyze papers that estimate smoking-attributable mortality in South America; and to provide an overview of the impact of smoking habit on mortality in this region.

## METHODS

We conducted a systematic review of the literature through following in general the indications of the Cochrane Handbook for systematic reviews of interventions. We report the methodology and results of the systematic review according to the PRISMA checklist [8], see Table S1 in the **Online Supplementary Document****.** To identify studies that estimated SAM, a search was conducted in the PubMed (MEDLINE), Embase, Latin American & Caribbean Health Sciences Literature (*Literatura Latinoamericana y del Caribe en Ciencias de la Salud/*LILACS) and *Biblioteca Virtual en Salud* (BVS) databases and the Google Scholar and Google search engines. The search criteria used were (tobacco OR smok* AND “mortality attribut*”) along with the MeSH terms (smoking AND mortality), and their combinations with the name of the region “South America” and each country (Argentina, Bolivia, Brazil, Chile, Colombia, Ecuador, Paraguay, Peru, Uruguay and Venezuela). In addition, we conducted a manual search and examination of other sources, based on the references cited in the papers retrieved. The designated search time limit was June 2020. Details of the search strategy used are presented in Appendix S2 in the **Online Supplementary Document**.

### Inclusion and exclusion criteria

We included all papers identified, regardless of the type of publication (including proceedings of congresses and conferences), as having been undertaken in South American countries in which SAM was estimated. No language restrictions were imposed, and simulation studies or economic projections were excluded.

### Study-selection

In a first step, titles and abstracts were separately reviewed by two members of the team, and the full text was then obtained for any paper that met the inclusion criteria.

Subsequently, the selected papers were read in full and the information entered on a data-extraction table (A.R, A.G.-O., M.P.-R), listing the variables that identified the studies (author, year of publication), the methodological aspects, and the results of the AM estimates. As regards the methodological aspects, the following data were obtained: study country or area; method of attribution, ie, prevalence- or non-prevalence-based; source and year of prevalence of tobacco use and, where applicable, the risks used and observed mortality (OM); year of the Surgeon General's Reports used as reference in the establishment of causal relationships [5,9,10]; causes of mortality analysed, year of attribution of mortality, and age groups of attribution of mortality. When it came to the results of the estimates, we extracted the total number of smoking-attributable deaths among men, among women and overall, and estimated the burden of attributable vs observed mortality. Any disagreements or discrepancies in interpretation of the data were settled by consensus (A.G.-O., M.P.-R).

### Ethical considerations

No Ethics Committee approval was required for the purposes of this review, due to the fact that the study relied on freely accessible, secondary databases; in this regard, the study poses no ethical risks for persons or the environment.

## RESULTS

**Figure 1:**
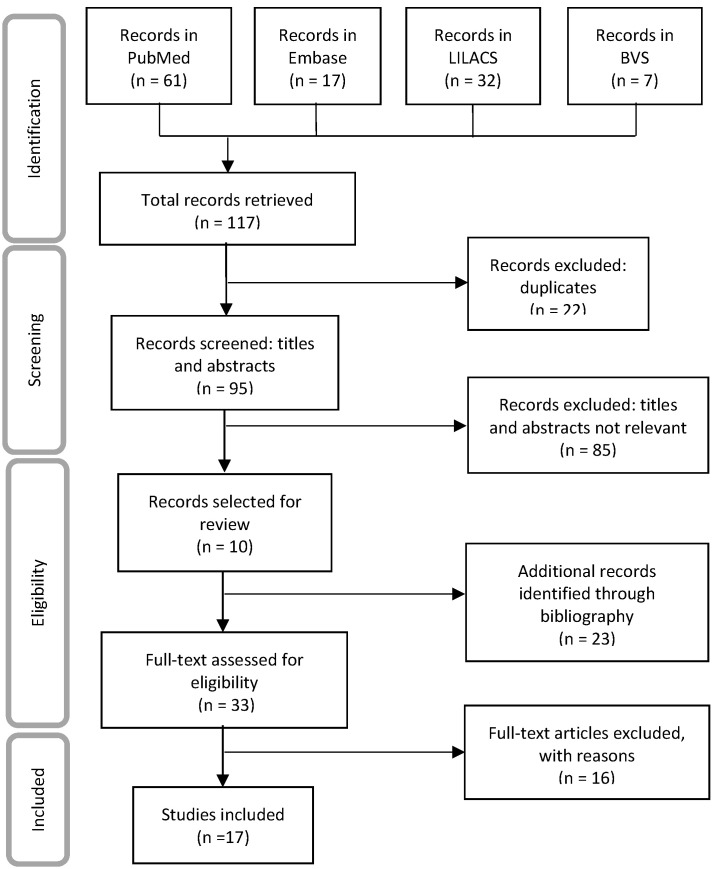
Flowchart of the included studies estimation smoking-attributable mortality in South America.

The search yielded 140 papers, 17 of which fulfilled the established inclusion criteria (8 scientific papers, 3 regional reports, 2 work documents, 2 local reports, 1 world report and 1 bulletin) and included 41 SAM estimates (**Figure 1**). The documents excluded were short communications without access to the full data or which reported OM data (3), economic models (6), mortality risk estimates (5), population attributable fractions (1), potential years of life lost or life expectancy (1).

**Figure 2:**
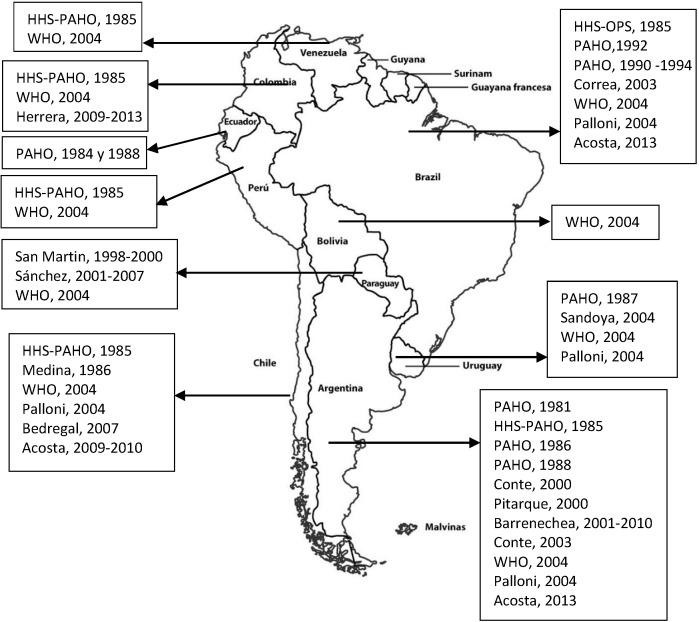
Estimations of the smoking-attributable mortality in South America (author, year of attribution of mortality). HHS – United States Department of Health and Human Services, PAHO – Pan American Health Organization, WHO – World Health Organization.

There were SAM estimates for all South American countries, with the earliest dating from 1981 for Argentina [11] and the most recent being for Argentina, Brazil and Colombia in 2013 [12,13] (**Table 1** and **Figure 2**). The countries with most estimates were Argentina with 11 [11,12,14-20], followed by Brazil with 8 [11,12,14,18,19,21,22] and Chile with 6 [11,12,18,19,23,24] (**Figure 2**). In 5 of the studies, SAM was estimated for more than one country [11,12,14,18,19], while others were limited to specific zones of the country, such as Tucuman in Argentina [20], 16 cities in Brazil [22], and the Caribbean region in Colombia [13]. Bolivia, Ecuador, Peru and Venezuela only had estimates drawn from reports issued by official bodies [11,14,18].

**Table 1:** Methodology applied in smoking-attributable mortality studies in South America and their results

Year of publication [Ref.]	Method	Source and year of prevalence	Source of risks	Year of OM	SG of reference*	Year of attribution	Age of estimate	% SAM			Causes‡	
**% Men†**	**% Women†**	**AM/OM**	**Not included**	**Additional**
**Argentina**												
1992 [14]	PB	Local and national surveys, § 1977, 1981	CPS-II	1981	1989	1981	55-64, ≥65	–	–	18.0	COPD	–
1992 [11]	NPB	Not applicable	CPS-II	1985	1964	1985	55-64, ≥65	90.7	9.3	6.8	–	–
1992 [14]	PB	Local and national surveys, § 1982, 1988	CPS-II	1986	1989	1986	55-64, ≥65	–	–	20.3	COPD	–
1992 [14]	PB	Local and national surveys, § 1990, 1991	CPS-II	1988	1989	1988	55-64, ≥65	–	–	14.8	COPD	–
2000 [15] and 2003 [16]	PB	National survey, 1999	CPS-II	2000	1997	2000	35-64, ≥65	71	29	15.7	Stomach cancer, AML	Arterial hypertension
2005 [17]	PB	National survey, 2004	CPS-II	2003	1997	2003	35-64, ≥65	69	31	15.1	–	Arterial hypertension
2012 [18] and 2015 [19]	NPB	Not applicable	CPS-II	2004	2004	2004	30-44, 45-59, 60-69, 70-79, ≥80	–	–	11.8	COPD	–
2016 [20]¶	PB	National survey, 2005 and 2009	CPS-II	2001	1997	2001-2010	35-64, ≥65	77.2	22.8	4.1	Cervical cancer, AML, atherosclerosis, other arterial disease	–
2019 [12]	PB	National survey, 2013	CPS-II	2012-2014	2014	2013	≥35	74.6	25.4	9.8	Liver cancer, colon cancer, rectal cancer, diabetes mellitus, TBC	–
**Bolivia**												
2012 [18]	NPB	Not applicable	CPS-II	2004	2004	2004	30-44, 45-59, 60-69, 70-79, ≥80	–	–	3.0	–	TBC, lower respiratory tract diseases
**Brazil**												
1992 [11]	NPB	Not applicable	CPS-II	1985	1964	1985	55-64, ≥65	81.2	18.8	2.2	–	–
1992 [14] and 2002 [21]	PB	National survey, 1989	CPS-II	1985	1992	1985	≥35	–	–	3.2	–	Other heart diseases, other vascular diseases; other respiratory diseases; all other ill-defined causes
2009 [22]¶	PB	National survey, 2002-2003	CPS-II	2003	2004	2003	35-64, ≥65	69.8	30.2	13.6	–	–
2012 [18] and 2015 [19]	NPB	Not applicable	CPS-II	2004	2004	2004	30-44, 45-59, 60-69, 70-79, ≥80	–	–	10.5	–	TBC, lower respiratory tract diseases
2019 [12]	PB	National survey, 2013	CPS-II	2012-2014	2014	2013	≥35	69.5	30.5	10.8	Liver cancer, colon cancer, rectal cancer, diabetes mellitus, TBC	–
**Chile**												
1992 [11]	NPB	Not applicable	CPS-II	1985	1964	1985	55-64, ≥65	77.8	22.2	4.7	–	–
1991 [23]	NPB	Not applicable	Others	1986	1982	1986	≥15	–	–	9.7	–	Arterial hypertension, TBC, chronic gastritis and peptic ulcer
2012 [18] and 2015 [19]	NPB	Not applicable	CPS-II	2004	2004	2004	30-44, 45-59, 60-69, 70-79, ≥80	–	–	9.6	–	TBC, lower respiratory tract diseases
2008 [24]	NPB	Not applicable	CPS-II	2004	2004	2007	20-44, 45-59, 60-74, ≥75	68.6	31.4	9.5	–	–
2019 [12]	PB	National survey, 2009-2010	CPS-II	2009-2011	2014	2009-2010	≥35	66.3	33.7	11.3	Liver cancer, colon cancer, rectal cancer, diabetes mellitus, TBC	–
**Colombia**												
1992 [11]	NPB	Not applicable	CPS-II	1985	1964	1985	≥35	64.6	35.4	1.8	–	–
2012 [18]	NPB	Not applicable	CPS-II	2004	2004	2004	30-44, 45-59, 60-69, 70-79, ≥80	–	–	8.3	–	TBC, lower respiratory tract diseases
2019 [13]¶	PB	National survey, 2010	CPS-II	2009, 2013	2004	2009-2013	35-64, ≥65	62.3	37.7	–	Ischaemic heart disease, rheumatic heart disease, other cardiac diseases and cardiopulmonary disease, CVD; artherosclerosis, aneurysm, influenza-pneumonia, COPD.	Prostate and breast cancer.
**Ecuador**												
1992 [14]	PB	Local and national surveys, ‖ 1954, 1988, 1990 and 1991	CPS-II	1984-1988	1989	1984 and 1988	45-54, 55-64, ≥65	–	–	10.0	–	–
**Paraguay**												
2006 [25]	PB	National survey, 2003	CPS-II	1998-2000	2004	1998-2000	35-65	65	35	13.4	Stomach cancer, AML	Arterial hypertension
2010 [26]	PB	National survey, 2003-2004	CPS-II	2001-2007	2004	2001-2007	35-64	76	24	12.0	Stomach cancer, AML	Arterial hypertension
2012 [18]	NPB	Not applicable	CPS-II	2004	2004	2004	30-44, 45-59, 60-69, 70-79, ≥80	–	–	6.5	–	TBC, lower respiratory tract diseases
**Peru**												
1992 [11]	NPB	Not applicable	CPS-II	1985	1964	1985	55-64, ≥65	100		0.2	–	–
2012 [18]	NPB	Not applicable	CPS-II	2004	2004	2004	30-44, 45-59, 60-69, 70-79, ≥80	–	–	3.5	–	TBC, lower respiratory tract diseases
**Uruguay**												
1992 [14]	PB	National survey, 1988	CPS-II	1987	1989	1897	35-64, ≥64	–	–	14.1	CVD	Cervical cancer, artherosclerosis, other cardiovascular diseases, pneumonias and other pulmonary diseases; perinatal diseases
2011 [27]	PB	National survey, 2010	CPS-II	2004	2004	2004	35-64, ≥65	72.3	27.7	14.8	–	–
2012 [18] and 2015 [19]	NPB	Not applicable	CPS-II	2004	2004	2004	30-44, 45-59, 60-69, 70-79, ≥80	–	–	13.4	–	TBC, lower respiratory tract diseases
**Venezuela**												
1992 [11]	NPB	Not applicable	CPS-II	1985	1964	1985	55-64, ≥65	66	34	2.4	–	–
2012 [18]	NPB	Not applicable	CPS-II	2004	2004	2004	30-44, 45-59, 60-69, 70-79, ≥80	–	–	8.8	–	TBC, lower respiratory tract diseases

OM – observed mortality, SG – Surgeon General, AM – attributable mortality, PB – prevalence-based, NPB – non-prevalence-based, CPS-II – Cancer Prevention Study II, DHHS – USA Department of Health and Human Services, AML – acute myeloid leukaemia, TBC – tuberculosis, CVD – cerebrovascular disease, COPD – chronic obstructive pulmonary disease.

*The year shown corresponds to the year of the Surgeon General's Report considered in studies to establish the diseases in respect of which a causal relationship with tobacco had been confirmed.

†Estimated proportion of deaths attributed to tobacco use.

‡Included in relation with the causes established by the Surgeon General. Causes are deemed to mean the diseases with which a causal relationship has been established, and the latter correspond to individual nosological entities (eg, stomach cancer) or groups of ICD-10 codes used by international convention (eg, lung refers to trachea, bronchi and lung).

§Joly, 1977; Alvarez-Herrera, 1981; Zarate in 1982; Gallup, 1988; Hospital de Pediatría (Paediatric Hospital), 1988; Maxwell, 1990; Misdorp, 1990c; Hayes, 1990; Costa de Robert, 1990; Catterberg y Asociados, 1991.

‖1954 Adult Tobacco Survey; 1988 Ministry of Health Survey; 1990 Quito Survey of Behaviour and Attitudes; 1991 CEIC/IESOP Survey.

¶The estimate is restricted to a specific area of the country.

The most commonly used method was prevalence-based (12 out of 17), with prevalences sourced from national surveys; in one study, prevalences were sourced from a tobacco-specific survey [27]. The risks applied were mainly drawn from the Cancer Prevention Study II [25] (16 out of 17). Observed mortality, which was drawn from vital statistics, had a lower age limit of 35 years and no upper limit in 13 of the 17 studies: in 2 studies the lower limit was set at 15 and 20 years respectively. The Surgeon General's Report most used as reference for the causes analysed was the 2004 edition [9] (**Table 1**).

Whereas studies published up to 1994 included 1 to 11 causes of death, those published after 1994 cited 2 to 19 causes of death. None included colon cancer, rectal cancer or diabetes mellitus (see Table S3 in the **Online Supplementary Document**).

SAM among adults in South America ranged from 184 (Peru, 1985) to 132 928 deaths (Brazil, 2013). Nineteen of the 41 estimates showed AM broken down by sex, with AM in all cases being highest in men. In Peru, AM estimates were restricted to men, and in Bolivia and Ecuador no estimates were shown with a breakdown by sex (**Table 1**).

The burden of AM vs OM varied among countries, and for countries with more than one estimate available it also changed with time, except in Uruguay where the impact appeared to remain stable. While Paraguay showed a decrease and Chile showed an increase in the burden of SAM, the remaining countries displayed no observably defined pattern (**Table 1**).

In 31 of the 37 estimates of AM which differentiated the results by reference to smoking-related disease groups, the greatest AM burden was attributed to the group of cardiovascular diseases. In terms of specific causes, ischaemic heart disease, lung cancer and COPD proved to be the leading specific causes of SAM.

## DISCUSSION

To our knowledge, this is the first systematic review of SAM in South America. There is at least one SAM estimate for each of the countries but 6 of the 10 countries that comprise the region have no estimates later than 2010. The most recent estimates are from 2013 for 3 of the 10 constituent countries. For Bolivia, Ecuador, Peru and Venezuela, the AM estimates are drawn from reports issued by the World Health Organisation (WHO) and Pan American Organisation Health (PAHO), with the characterisation of data sources being very limited and thus not allowing for in-depth analysis of the estimates obtained and their potential limitations.

The periodicity of the estimates is unforeseeable, which may be due to the fact that until now South America has had other health priorities. These priorities included things such as non-communicable diseases (cardiovascular diseases, cancer, diabetes, and chronic respiratory diseases) that account for 73.2% of all deaths [26], but did not extend to the study of the epidemiology of the risk factors for such diseases.

Although there is no consensus about the method of choice for estimating SAM [7], most of the studies were found to have applied the prevalence-based method. The use of this method is frequent in the USA and Europe, where smoking prevalence data are regularly available and are drawn from national health studies that are conducted in accordance with international guidelines. However, things are not quite the same in South America, where the availability of prevalence data are limited, or the age groups for which such data are available differ from those established by international consensus; hence, two studies set upper age limits in the estimate of prevalence at 64 years [28,29] which render it impossible to estimate AM in persons over the age of 64 years. Furthermore, in the prevalence of non-smokers cited by some surveys, there is no way of distinguishing the ex-smokers [30]. This gives rise to an underestimate of AM.

When it comes to observed mortality, its quality must be assessed, since there are problems of under-registration and miscoding [12,31]. Evaluation of the performance of the vital statistics registration systems from which the observed mortality data were drawn ranges from 0.217 in Bolivia to 0.913 in Venezuela, which indicates that vital statistics in Bolivia account for only 21.7% vs 91.3% in Venezuela. Across the period 1980-2012, the quality and general utility of death statistics data in Latin America and the Caribbean had a mean score of 0.765 over 1 (range 0.617 to 0.913) [32,33].

In South America there are no studies from which one can ascertain smokers’ and ex-smokers’ excess risk of dying from smoking-related diseases, ie, relative risks of mortality. It is precisely because of this that, in all but one of the studies included [23], the risk estimates are drawn from the follow-up of the Cancer Prevention Study II cohort [25]. This US cohort study provides the best evidence available to date, though it should be borne in mind here that smoking histories and, by extension, risks are very different in South America and the USA [2].

With respect to the causes of death analysed, the evidence showed that with the passage of time, more causes were gradually added in AM estimation studies, a development in line with the Surgeon General’s practice of associating and confirming the causal relationship between other diseases and smoking habit, essentially via the reports issued for the years 1964 (8 causes), 1989 (10 causes), 2004 (18 causes) and 2014 (22 causes) [5]. Yet, despite the fact that studies were found to have been published over the period 2014-2019 [12,13], none of them analysed SAM with the 22 causes confirmed in the 2014 Surgeon General's Report.

A number of studies did not report AM by sex [14,18,19,21,23], something that limits the analysis in terms of how the smoking epidemic evolved by reference to this characteristic. This has a negative impact on the possibility of having a source of reference for the introduction, implementation and evaluation of smoking policies. SAM has been most closely studied in Argentina, Brazil and Chile, where smoking control policies have been progressively implemented since 1980 [12,34]. Hence, according to the WHO’s most recent follow-up report on the global smoking epidemic in South America, published in 2019, Brazil is the only country to have adopted all the MPOWER measures at the highest level [35]. Moreover, this country, along with Uruguay, are the regional leaders in tobacco control [12,34]. For their part, Argentina and Chile have implemented at least 4 of the MPOWER measures at the highest level of application [34]. It is noteworthy that, after the publication of the WHO Framework Convention on Tobacco Control in 2003 and the subsequent implementation of the MPOWER package, there has been no increase in the publication of studies that estimate SAM.

The disease group to which most mortality is attributed by the majority of countries is cardiovascular diseases. This mortality pattern is different to that of European countries, where in terms of attributable mortality -overall and in men- tumours exceed circulatory diseases when the burden of SAM is analysed [36]. These differences may be due, not only to insufficient control of the main risk factors, which, in addition to tobacco, influence the generation of cardiovascular diseases such as hypertension, hypercholesterolaemia and sedentarism, among others, but also to the need for improvements in the early care of acute processes, such as infarction and cerebrovascular diseases [37].

This study has a series of limitations, including those linked to the difficulty of identifying the data sources used for the AM estimates in some studies. Hence, there may have been some omissions in the extraction of data. In addition, the changeover from the International Classification of Diseases 9th to 10th revision (ICD-9 to ICD-10) in the coding of OM hindered the comparison of the estimates [5,11]. By way of advantages, stress should be laid on the systematic review design per se which, moreover, employed an exhaustive search strategy, thereby allowing for the inclusion of all available studies and exhaustive data-collection.

In conclusion, SAM estimates have been made for all South American countries. Despite the methodological differences between studies, most of these countries show the highest SAM burden to be associated with cardiovascular diseases. In many cases the available estimates are obsolete, meaning that there is a need for further studies to update the impact of smoking habit on most South American countries. Surveillance systems should be strengthened or implemented using data that are standardized, comparable (risks used, age groups analysed, causes included), representative at a national level, and regularly published on tobacco use and its impact on the population. It is vital that public administrations, health professionals and the public are aware of the impact of tobacco on mortality, since this would help strengthen the measures required to reduce its effects on health. AM estimates would also help with decision-making targeted at evaluating and enhancing the impact of smoking control policies [38,39].

## Additional material

Online Supplementary Document

## References

[1] Davey Smith C Egger M The first reports on smoking and lung cancer: why are they consistently ignored? Bull World Health Organ. 2005;83:799-800. 16283059 PMC2626411

[2] The Tobacco AtlasAvailable: www.tobaccoatlas.org. Accessed: 25 November 2019.

[3] World Health Organization. WHO report on the global tobacco epidemic, 2008: the MPOWER package. Geneva: WHO Library Cataloguing-in-Publication Data; 2008.

[4] Organización Panamericana de la Salud. Informe sobre el control del tabaco en la Región de las Américas, 2018. Washington, DC; OPS; 2018.

[5] U.S. Department of Health and Human Services. The Health Consequences of Smoking: 50 Years of Progress. A Report of the Surgeon General. The Health Consequences of Smoking-50 Years of Progress: A Report of the Surgeon General. Atlanta, GA: U.S. Department of Health and Human Services, Centers for Disease Control and Prevention, National Center for Chronic Disease Prevention and Health Promotion, Office on Smoking and Health; 2014.

[6] Peto R Lopez AD Boreham J Thun M Heath C Doll R Mortality from smoking worldwide. Br Med Bull. 1996;52:12-21. 10.1093/oxfordjournals.bmb.a011519 8746293

[7] Pérez-Ríos M Montes A Methodologies used to estimate tobacco-attributable mortality: a review. BMC Public Health. 2008;8:22. 10.1186/1471-2458-8-22 18211696 PMC2262075

[8] Moher D Liberati A Tetzlaff J Altman DG PRISMA Group. Preferred reporting items for systematic reviews and meta-analyses: The PRISMA statement. BMJ. 2009;339:b2535. 10.1136/bmj.b2535 19622551 PMC2714657

[9] U.S. Department of Health and Human Services. The Health Consequences of Smoking: A Report of the Surgeon General. Atlanta (GA): Centers for Disease Control and Prevention (US); 2004.

[10] U.S. Department of Health Education and Welfare. Smoking and Health: report of the advisory committee to the Surgeon General of the public health service. Washington, D.C.: Superintendent of documents, U.S. Government Printing Office; 1964.

[11] Departamento de Salud y Servicios Sociales de los Estados Unidos de América. Tabaquismo y salud en las Américas. Atlanta, Georgia: Departamento de Salud y Servicios Sociales (HHS) de los Estados Unidos de América, Servicio de Salud Pública, Centros para el Control de Enfermedades, Centro Nacional para la Prevención de Enfermedades Crónicas y Promoción de la Salud, Oficina de Tabaquismo y Salud; 1992.

[12] Acosta LD Molinatti F Peláez E Comparison of mortality attributable to tobacco in selected Latin American countries. Poblac Salud Mesoam. 2019;16:1-20.

[13] Herrera-Pamplona K Cogollo-Milanés Z Alvis-Estrada RL Mortalidad por cáncer asociado al consumo de cigarrillo en el Caribe Colombiano, 2009-2013. Rev Fac Nac Salud Pública. 2019;37:116-24. 10.17533/udea.rfnsp.v37n2a13

[14] Organización Panamericana de la Salud. Tabaco o salud: situación en las Américas. Washington, D.C: Catalogación por la Biblioteca de la OPS; 1992.

[15] Pitarque R, Perel P, Sánchez G. Mortalidad anual atribuible al tabaco en Argentina, año 2000: proyecto financiado por el Programa Vigi+A. Buenos Aires; 2000.

[16] Conte Grand M, Perel P, Pitarque R, Sánchez G. Estimación del costo económico en Argentina de la mortalidad atribuible al tabaco en adultos. Buenos Aires; 2003.

[17] Conte Grand M. Estimación actualizada del costo económico en Argentina de la mortalidad atribuible al tabaco en Adultos. Buenos Aires; 2005.

[18] World Health Organization. WHO global report: mortality attributable to tobacco. Geneva: WHO Library Cataloguing-in-Publication Data; 2012.

[19] Palloni A Novak B Pinto-Aguirre G The enduring effects of smoking in Latin America. Am J Public Health. 2015;105:1246-53. 10.2105/AJPH.2014.302420 25880938 PMC4431107

[20] Barrenechea GG Cali RS Smoking-attributable mortality in Tucumán, Argentina 2001-2010. Medicina (B Aires). 2016;76:287-93. 27723616

[21] Organización Panamericana de la Salud. La salud en las Américas, Edición de 2002, Volumen I. Washington, D.C.: Catalogación por la Biblioteca de la OPS; 2002.

[22] Corrêa PC Barreto SM Passos VM Smoking-attributable mortality and years of potential life lost in 16 Brazilian capitals, 2003: A prevalence-based study. BMC Public Health. 2009;9:206. 10.1186/1471-2458-9-206 19558658 PMC2711948

[23] Médina Lois E Kaempffer A Tabaquismo y salud en Chile. Bol Sanit Panam. 1991;111:112-21. 1834082

[24] Bedregal P, Margozzini P, González C, Aguilera X, González C, Rajs D, et al. Informe final estudio de carga de enfermedad y carga atribuible. Santiago de Chile: Ministerio de Salud de Chile. Subsecretaría de Salud Pública; 2008.

[25] Thun MJ Carter BD Feskanich D Freedman ND Prentice R Lopez AD 50-Year trends in smoking-related mortality in the United States. N Engl J Med. 2013;368:351-64. 10.1056/NEJMsa1211127 23343064 PMC3632080

[26] Organización Panamericana de la Salud. Enfermedades no transmisibles: hechos y cifras. Washington D.C.: OPS; 2019.

[27] Sandoya E Bianco E Mortalidad por tabaquismo y por humo de segunda mano en Uruguay. Rev Urug Cardiol. 2011;26:201-6.

[28] San Martín V Gamarra de Cáceres G Mortalidad atribuible al consumo de tabaco durante los años 1998, 1999 y 2000 en Paraguay. Mem Inst Investig Cienc Salud. 2006;4:15-9.

[29] Sánchez C San Martín V Mortalidad atribuible al tabaquismo durante los años 2001-2007 en Paraguay. Rev Parag Epidemiol. 2010;1:27-32.

[30] Epidat 4: Ayuda de estimación de la mortalidad atribuida. Available: https://www.sergas.es/Saude-publica/Documents/1897/Ayuda_Epidat4_Mortalidad_atribuida_Octubre2014.pdf. Accessed: 25 November 2019.

[31] Los datos demográficos: alcances, limitaciones y métodos de evaluación. Available: https://repositorio.cepal.org/handle/11362/37145 Accessed: 11 February 2020.

[32] Lopez AD McLaughlin D Richards N Reducing ignorance about who dies of what: Research and innovation to strengthen CRVS systems. BMC Med. 2020;18:58. 10.1186/s12916-020-01526-9 32146906 PMC7061482

[33] Phillips DE Lozano R Naghavi M Atkinson C Gonzalez-Medina D Mikkelsen L A composite metric for assessing data on mortality and causes of death: the vital statistics performance index. Popul Health Metr. 2014;12:14. 10.1186/1478-7954-12-14 24982595 PMC4060759

[34] Blanco A Sandoval RC Martínez-López L de Betânia Caixeta R Diez años del Convenio Marco de la OMS para el Control del Tabaco: Avances en las Américas. Salud Publica Mex. 2017;59 supl 1:s117-25. 10.21149/8682 28658460

[35] World Health Organization. WHO report on the global tobacco epidemic, 2019: offer help to quit tobacco use. Geneva: World Health Organization; 2019.

[36] Pérez-Ríos M Schiaffinoc A Montes A Fernández E López MJ Martínez-Sánchez JM Mortalidad atribuible al consumo de tabaco en España 2016. Arch Bronconeumol. 2020;56:559-63. 10.1016/j.arbres.2019.11.021 35373765

[37] Marmot M Bell R Social determinants and non-communicable diseases: time for integrated action. BMJ. 2019;364:l251. 10.1136/bmj.l251 30692093 PMC6348404

[38] Organización Mundial de la Salud. Convenio marco de la OMS para el Control del Tabaco. Ginebra: Catalogación por la Biblioteca de la OMS; 2003.

[39] GBD 2015 Tobacco Collaborators Smoking prevalence and attributable disease burden in 195 countries and territories, 1990-2015: A systematic analysis from the Global Burden of Disease Study 2015. Lancet. 2017;389:1885-906. 10.1016/S0140-6736(17)30819-X 28390697 PMC5439023

