# Supplementary material for: Smoking-attributable mortality in South America: A systematic review
Source: J Glob Health. 2021 Mar 27;11:04014. doi: 10.7189/jogh.11.04014 (PMC8005314; doi:10.7189/jogh.11.04014)
Supplement: Online Supplementary Document [file jogh-11-04014-s001.pdf]

Table S1
PRISMA 2009 Checklist

Section/topic	#	Checklist item	Reported on page #
**TITLE**			
Title	1	Identify the report as a systematic review, meta-analysis, or both.	1
**ABSTRACT**			
Structured summary	2	Provide a structured summary including, as applicable: background; objectives; data sources; study eligibility criteria, participants, and interventions; study appraisal and synthesis methods; results; limitations; conclusions and implications of key findings; systematic review registration number.	1, 2
**INTRODUCTION**			
Rationale	3	Describe the rationale for the review in the context of what is already known.	3
Objectives	4	Provide an explicit statement of questions being addressed with reference to participants, interventions, comparisons, outcomes, and study design (PICOS).	NA
**METHODS**			
Protocol and registration	5	Indicate if a review protocol exists, if and where it can be accessed (e.g., Web address), and, if available, provide registration information including registration number.	NA
Eligibility criteria	6	Specify study characteristics (e.g., PICOS, length of follow-up) and report characteristics (e.g., years considered, language, publication status) used as criteria for eligibility, giving rationale.	5,6
Information sources	7	Describe all information sources (e.g., databases with dates of coverage, contact with study authors to identify additional studies) in the search and date last searched.	5
Search	8	Present full electronic search strategy for at least one database, including any limits used, such that it could be repeated.	5 Supplementary table 1
Study selection	9	State the process for selecting studies (i.e., screening, eligibility, included in systematic review, and, if applicable, included in the meta-analysis).	5,6
Data collection process	10	Describe method of data extraction from reports (e.g., piloted forms, independently, in duplicate) and any processes for obtaining and confirming data from investigators.	6

Data items	11	List and define all variables for which data were sought (e.g., PICOS, funding sources) and any assumptions and simplifications made.	6
Risk of bias in individual studies	12	Describe methods used for assessing risk of bias of individual studies (including specification of whether this was done at the study or outcome level), and how this information is to be used in any data synthesis.	NA
Summary measures	13	State the principal summary measures (e.g., risk ratio, difference in means).	6
Synthesis of results	14	Describe the methods of handling data and combining results of studies, if done, including measures of consistency (e.g., I^2) for each meta-analysis.	NA
**Section/topic**	**#**	**Checklist item**	**Reported on page #**
Risk of bias across studies	15	Specify any assessment of risk of bias that may affect the cumulative evidence (e.g., publication bias, selective reporting within studies).	NA
Additional analyses	16	Describe methods of additional analyses (e.g., sensitivity or subgroup analyses, meta-regression), if done, indicating which were pre-specified.	NA
**RESULTS**			
Study selection	17	Give numbers of studies screened, assessed for eligibility, and included in the review, with reasons for exclusions at each stage, ideally with a flow diagram.	7<br>Figure 1
Study characteristics	18	For each study, present characteristics for which data were extracted (e.g., study size, PICOS, follow-up period) and provide the citations.	7,8<br>Table 1 and Figure 2
Risk of bias within studies	19	Present data on risk of bias of each study and, if available, any outcome level assessment (see item 12).	NA
Results of individual studies	20	For all outcomes considered (benefits or harms), present, for each study: (a) simple summary data for each intervention group (b) effect estimates and confidence intervals, ideally with a forest plot.	7,8
Synthesis of results	21	Present results of each meta-analysis done, including confidence intervals and measures of consistency.	NA
Risk of bias across studies	22	Present results of any assessment of risk of bias across studies (see Item 15).	NA
Additional analysis	23	Give results of additional analyses, if done (e.g., sensitivity or subgroup analyses, meta-regression [see Item 16]).	NA
**DISCUSSION**			
Summary of evidence	24	Summarize the main findings including the strength of evidence for each main outcome; consider their relevance to key groups (e.g., healthcare providers, users, and policy makers).	9

Limitations	25	Discuss limitations at study and outcome level (e.g., risk of bias), and at review-level (e.g., incomplete retrieval of identified research, reporting bias).	12
Conclusions	26	Provide a general interpretation of the results in the context of other evidence, and implications for future research.	12
**FUNDING**			
Funding	27	Describe sources of funding for the systematic review and other support (e.g., supply of data); role of funders for the systematic review.	Title page

Source: Moher D, Liberati A, Tetzlaff J, Altman DG, The PRISMA Group (2009). Preferred Reporting Items for Systematic Reviews and Meta-Analyses: The PRISMA Statement. PLoS Med 6(7): e1000097. doi:10.1371/journal.pmed1000097

Appendix S2

Database: EMBASE. Epub Ahead of Print or formally published
Publication years: <1966 - 2020
Search Strategies:
....
No. Query _ Results
#28. #1 AND #16 AND #17 (1)
#27. #1 AND #15 AND #17 (2)
#26. #1 AND #14 AND #17
#25. #1 AND #13 AND #17 (1)
#24. #1 AND #12 AND #17
#23. #1 AND #11 AND #17 (1)
#22. #1 AND #10 AND #17 (3)
#21. #1 AND #9 AND #17 (4)
#20. #1 AND #8 AND #17
#19. #1 AND #7 AND #17 (2)
#18. #1 AND #6 AND #17 (1)
#17. #2 OR #3 OR #4 (618,958)
#16. venezuela (31,698)
#15. uruguay (21,656)
#14. peru (31,679)
#13. paraguay (6,590)
#12. ecuador (14,079)
#11. colombia (65,151)
#10. chile (99,067)
#9. brazil (652,335)
#8. bolivia (6,625)
#7. argentina (202,743)
#6. 'south america' (26,969)
#5. mortality (1,568,051)
#4. smoking (498,342)
#3. smok* (551,347)
#2. tobacco (169,220)
#1. 'mortality attribut*' (2,058)
....

Table S3
Causes of death with tobacco causal association established according to the Surgeon General. Correspondence with the codes of the International Classification of Diseases 10th revision (ICD-10)

Causes of death*	Year of confirmation	ICD-10 code
**Cancer**		
Trachea- bronchus-lung	1964 (1967: women)	C33-34
Other cancers		
Lip-Mouth-Pharynx	1982	C00-14
Esophagus	1982	C15
Stomach	2001	C16
Pancreas	1990	C25
Larynx	1980	C32
Cervix uteri	2001	C53
Kidney and renal pelvis	1982	C64-65
Rinary bladder	1990	C67
Liver	2014	C22
Colorectal	2014	C18-20
Acute myeloid leukemia	1990	C92
**Cardiometabolic diseases**		
Coronary heart diseases	1979	I20-25
Other heart diseases		
Rheumatic heart diseases	1983	I00-02, I05-09
Other forms of heart disease and cardiopulmonary disease	1983 - 1990	I26-51
Cerebrovascular diseases	1989	I60-69
Diseases of arteries, arterioles and capillaries		
Atherosclerosis	1983	I70
Aortic aneurysm and other arterial disease	1983	I71-78
Diabetes mellitus	2014	E10-14
**Respiratory diseases**		
Chronic obstructive pulmonary disease	1964	J40-44
Influenza, pneumonia	1990	J09-18
Tuberculosis	2014	A15-A19

*In some cases, causes are understood to be groupings of ICD-10 codes used by international convention (example: lung refers to trachea, bronchi and lung) and in other cases to individual nosological entities (example: stomach cancer).

Source: own elaboration on the basis of references 5, 9, 10.
